# Asthma

**DOI:** 10.1186/s13223-018-0279-0

**Published:** 2018-09-12

**Authors:** Jaclyn Quirt, Kyla J. Hildebrand, Jorge Mazza, Francisco Noya, Harold Kim

**Affiliations:** 1 McMaster University, Hamilton, ON Canada; 2 University of British Columbia, Vancouver, BC Canada; 3 Western University, London, ON Canada; 4 McGill University, Montreal, QC Canada

## Abstract

Asthma is the most common respiratory disorder in Canada. Despite significant improvement in the diagnosis and management of this disorder, the majority of Canadians with asthma remain poorly controlled. In most patients, however, control can be achieved through the use of avoidance measures and appropriate pharmacological interventions. Inhaled corticosteroids (ICS) represent the standard of care for the majority of patients. Combination ICS/long-acting beta_2_-agonist inhalers are preferred for most adults who fail to achieve control with ICS therapy. Biologic therapies targeting immunoglobulin E or interleukin-5 are recent additions to the asthma treatment armamentarium and may be useful in select cases of difficult to control asthma. Allergen-specific immunotherapy represents a potentially disease-modifying therapy for many patients with asthma, but should only be prescribed by physicians with appropriate training in allergy. In addition to avoidance measures and pharmacotherapy, essential components of asthma management include: regular monitoring of asthma control using objective testing measures such as spirometry, whenever feasible; creation of written asthma action plans; assessing barriers to treatment and adherence to therapy; and reviewing inhaler device technique. This article provides a review of current literature and guidelines for the appropriate diagnosis and management of asthma in adults and children.

## Background

Asthma remains the most common chronic respiratory disease in Canada, affecting approximately 10% of the population [1]. It is also the most common chronic disease of childhood [2]. Although asthma is often believed to be a disorder localized to the lungs, current evidence indicates that it may represent a component of systemic airway disease involving the entire respiratory tract, and this is supported by the fact that asthma frequently coexists with other atopic disorders, particularly allergic rhinitis [3].

Despite significant improvements in the diagnosis and management of asthma over the past decade, as well as the availability of comprehensive and widely-accepted national and international clinical practice guidelines for the disease, asthma control in Canada remains suboptimal. Results from the Reality of Asthma Control in Canada study suggest that over 50% of Canadians with asthma have uncontrolled disease [4]. Poor asthma control contributes to unnecessary morbidity, limitations to daily activities and impairments in overall quality of life [1].

This article provides an overview of diagnostic and therapeutic guideline recommendations from the Global Initiative for Asthma (GINA) and the Canadian Thoracic Society and as well as a review of current literature related to the pathophysiology, diagnosis, and appropriate treatment of asthma.

## Definition

Asthma is defined as a chronic inflammatory disease of the airways. The chronic inflammation is associated with airway hyperresponsiveness (an exaggerated airway-narrowing response to specific triggers such as viruses, allergens and exercise) that leads to recurrent episodes of wheezing, breathlessness, chest tightness and/or coughing that can vary over time and in intensity. Symptom episodes are generally associated with widespread, but variable, airflow obstruction within the lungs that is usually reversible either spontaneously or with appropriate asthma treatment such as a fast-acting bronchodilator [5].

## Epidemiology

The 2003 Canadian Community Health Survey found that 8.4% of the Canadian population ≥ 12 years of age had been diagnosed with asthma, with the prevalence being highest among teens (> 12%) [6]. Between 1998 and 2001, close to 80,000 Canadians were admitted to hospital for asthma, and hospitalization rates were highest among young children and seniors. However, the survey also found that mortality due to asthma has fallen sharply since 1985. In 2001, a total of 299 deaths were attributed to asthma. Seven of these deaths occurred in persons under 19 years of age, while the majority (62%) occurred in those over 70 years of age [6].

More recent epidemiological evidence suggests that that the prevalence of asthma in Canada is rising, particularly in the young population. A population-based cohort study conducted in Ontario found that the age- and sex-standardized asthma prevalence increased from 8.5% in 1996 to 13.3% in 2005, a relative increase of 55% [7]. The age-standardized increase in prevalence was greatest in adolescents and young adults compared with other age groups, and the gender-standardized increase in prevalence was greater in males compared with females. Compared with females, males experienced higher increases in prevalence in adolescence and young adulthood and lower increases at age 70 years or older.

Another recent study of over 2800 school-aged children in Toronto that assessed parental reports of asthma by questionnaire found the prevalence of asthma to be approximately 16% in this young population [8]. The results of these studies suggest that effective clinical and public health strategies are needed to prevent and manage asthma in the Canadian population.

## Pathophysiology and etiology

Asthma is associated with T helper cell type-2 (Th2) immune responses, which are typical of other atopic conditions. Asthma triggers may include allergic (e.g., house dust mites, cockroach residue, animal dander, mould, and pollens) and non-allergic (e.g., viral infections, exposure to tobacco smoke, cold air, exercise) stimuli, which produce a cascade of events leading to chronic airway inflammation. Elevated levels of Th2 cells in the airways release specific cytokines, including interleukin (IL)-4, IL-5, IL-9 and IL-13, and promote eosinophilic inflammation and immunoglobulin E (IgE) production. IgE production, in turn, triggers the release of inflammatory mediators, such as histamine and cysteinyl leukotrienes, that cause bronchospasm (contraction of the smooth muscle in the airways), edema, and increased mucous secretion, which lead to the characteristic symptoms of asthma [5, 9].

The mediators and cytokines released during the early phase of an immune response to an inciting trigger further propagate the inflammatory response (late-phase asthmatic response) that leads to progressive airway inflammation and bronchial hyperreactivity [9]. Over time, the airway remodeling that occurs with frequent asthma exacerbations leads to greater lung function decline and more severe airway obstruction [10]. This highlights the importance of frequent assessment of asthma control and the prevention of exacerbations.

Evidence suggests that there may be a genetic predisposition for the development of asthma. Several chromosomal regions associated with asthma susceptibility have been identified, such as those related to the production of IgE antibodies, expression of airway hyperresponsiveness, and the production of inflammatory mediators. However, further study is required to determine specific genes involved in asthma as well as the gene-environment interactions that may lead to expression of the disease [5, 9].

An extensive literature review undertaken as part of the development of the Canadian Healthy Infant Longitudinal Development (CHILD) study (an ongoing multicentre national observational study) examined risk factors for the development of allergy and asthma in early childhood [11]. Prenatal risk factors linked to early asthma development include: maternal smoking, use of antibiotics and delivery by caesarean section. With respect to prenatal diet and nutrition, a higher intake of fish or fish oil during pregnancy, and higher prenatal vitamin E and zinc levels have been associated with a lower risk of development of wheeze in young children. Later in childhood, risk factors for asthma development include: allergic sensitization (particularly house dust mite, cat and cockroach allergens), exposure to environmental tobacco smoke, breastfeeding (which may initially protect and then increase the risk of sensitization), decreased lung function in infancy, antibiotic use and infections, and gender. Future results from CHILD may help further elucidate risk factors for asthma development.

## Asthma phenotypes

Although asthma has long been considered a single disease, recent studies have increasingly focused on its heterogeneity [12]. The characterization of this heterogeneity has led to the concept that asthma consists of various “phenotypes” or consistent groupings of characteristics. Using a hierarchical cluster analysis of subjects from the Severe Asthma Research Program (SARP), Moore and colleagues [13] have identified five distinct clinical phenotypes of asthma which differ in lung function, age of asthma onset and duration, atopy and sex.

In children with asthma, three wheeze phenotypes have been identified: (1) transient early wheezing; (2) non-atopic wheezing; and (3) IgE-mediated (atopic) wheezing [14]. The transient wheezing phenotype is associated with symptoms that are limited to the first 3–5 years of life; it is not associated with a family history of asthma or allergic sensitization. Risk factors for this phenotype include decreased lung function that is diagnosed before any respiratory illness has occurred, maternal smoking during pregnancy, and exposure to other siblings or children at daycare centres. The non-atopic wheezing phenotype represents a group of children who experience episodes of wheezing up to adolescence that are not associated with atopy or allergic sensitization. Rather, the wheezing is associated with a viral respiratory infection [particularly with the respiratory syncytial virus (RSV)] experienced in the first 3 years of life. Children with this phenotype tend to have milder asthma than the atopic phenotype. IgE-mediated (atopic) wheezing (also referred to as the “classic asthma phenotype”) is characterized by persistent wheezing that is associated with atopy, early allergic sensitization, significant loss of lung function in the first years of life, and airway hyperresponsiveness.

Classifying asthma according to phenotypes provides a foundation for improved understanding of disease causality and the development of more targeted and personalized approaches to management that can lead to improved asthma control [13]. Research on the classification of asthma phenotypes and the appropriate treatment of these phenotypes is ongoing.

## Diagnosis

The diagnosis of asthma involves a thorough medical history, physical examination, and objective assessments of lung function in those ≥ 6 years of age (spirometry preferred, both before and after bronchodilator) to document variable expiratory airflow limitation and confirm the diagnosis (see Table 1). Bronchoprovocation challenge testing and assessing for markers of airway inflammation may also be helpful for diagnosing the disease, particularly when objective measurements of lung function are normal despite the presence of asthma symptoms [5, 15, 16].

**Table 1:** Diagnosis of asthma based on medical history, physical examination and objective measurements [5, 15, 16]

**Medical history**	
• Assess for classic symptoms of asthma: − Wheezing − Breathlessness − Chest tightness − Cough (with our without sputum) • Assess for symptom patterns suggestive of asthma: − Recurrent/episodic − Occur/worsen at night or early in the morning − Occur/worsen upon exposure to allergens (e.g., animal dander, pollen, dust mites) or irritants (e.g., exercise, cold air, tobacco smoke, infections) − Respond to appropriate asthma therapy • Assess for family or personal history of atopic disease (particularly allergic rhinitis)	
**Physical examination** • Examine for wheezing on auscultation • Examine upper respiratory tract and skin for signs of other atopic conditions	
** Objective measures for confirming variable expiratory airflow limitation (spirometry preferred)** • Documented airflow limitation: ▪ *Diagnostic criteria:* at least once during diagnostic process when FEV_1_ is low, confirm that FEV_1_/FVC is reduced (normally > 0.75–0.80 in adults, > 0.90 in children)	
	AND
• Documented excessive variability in lung function using one or more of the tests below (the greater the variations, or the more occasions excess variation is seen, the more confident the diagnosis):	
	Diagnostic criteria
▪ Positive bronchodilator (BD) reversibility test^a^ (more likely to be positive if BD is withheld before test: SABA ≥ 4 h, LABA ≥ 15 h)	*→ Adults* increase in FEV_1_ of > 12% and > 200 mL from baseline, 10–15 min after 200–400 μg albuterol or equivalent (greater confidence if increase is > 15% and > 400 mL) *→ Children* increase in FEV_1_ of > 12% predicted
▪ Excessive variability in twice-daily PEF over 2 weeks^a^	*→ Adults* average daily diurnal PEF variability > 10%^b^ *→ Children* average daily diurnal PEF variability > 13%^b^
▪ Significant increase in lung function after 4 weeks of anti-inflammatory treatment	*→ Adults* increase in FEV_1_ by > 12% and > 200 mL (or PEF^c^ by > 20%) from baseline after 4 weeks of treatment, outside respiratory infections
▪ Positive exercise challenge test^a^	*→ Adults* fall in FEV_1_ of > 10% and > 200 mL from baseline *→ Children* fall in FEV_1_ of > 12% predicted, or PEF > 15%
▪ Positive bronchial challenge test (usually only performed in adults)	*→* Fall in FEV_1_ from baseline of ≥ 20% with standard doses of methacholine or histamine, or ≥ 15% with standardized hyperventilation, hypertonic saline or mannitol challenge
▪ Excessive variation in lung function between visits (less reliable)^a^	*→ Adults* variation in FEV_1_ of > 12% and > 200 mL between visits, outside of respiratory infections *→ Children* variation in FEV_1_ of > 12% or > 15% in PEF^c^ between visits (may include respiratory infections)
**Allergy testing** • Perform skin tests to assess allergic status and identify possible triggers	

*FVC* forced vital capacity, *FEV*_*1*_ forced expiratory volume in 1 s, *PEF* peak expiratory flow (highest of three readings), *BD* bronchodilator (short-acting SABA or rapid-acting LABA), *LABA* long-acting beta_2_-agonist, *SABA* short-acting beta_2_-agonist

^a^These tests can be repeated during symptoms or in the early morning

^b^Daily diurnal PEF variability is calculated from twice daily PEF as ([day’s highest minus day’s lowest]/mean of day’s highest and lowest), and averaged over 1 week

^c^For PEF, use the same meter each time, as PEF may vary by up to 20% between different meters. BD reversibility may be lost during severe exacerbations or viral infections. If bronchodilator reversibility is not present at initial presentation, the next step depends on the availability of other tests and the urgency of the need for treatment. In a situation of clinical urgency, asthma treatment may be commenced and diagnostic testing arranged within the next few weeks, but other conditions that can mimic asthma should be considered, and the diagnosis of asthma confirmed as soon as possible

The importance of labeling asthma properly in children and preschoolers cannot be overemphasized since recurrent preschool wheezing has been associated with significant morbidity that can impact long-term health [17]. According to a recent position statement by the Canadian Paediatric Society and the Canadian Thoracic Society, asthma can be appropriately diagnosed as such in children 1–5 years of age, and terms that denote either a suggestive pathophysiology (e.g., ‘bronchospasm’ or ‘reactive airway disease’) or vague diagnoses (e.g., ‘wheezy bronchitis’ or ‘happy wheezer’) should be abandoned in medical records [17].

### Medical history

Important questions to ask when taking the medical history of patients with suspected asthma are summarized in Table 2. The diagnosis of asthma should be suspected in patients with recurrent cough, wheeze, chest tightness and/or shortness of breath. Symptoms that are variable, occur upon exposure to triggers such as allergens or irritants, that often worsen at night and that respond to appropriate asthma therapy are strongly suggestive of asthma [5, 16]. Alternative causes of suspected asthma symptoms should be excluded (see “Differential diagnosis” section in this article).

**Table 2:** Key questions to ask when taking the medical history of patients with suspected asthma

• Asthma symptoms (cough, wheeze, increased work of breathing)?
• Age of onset of symptoms?
• Timing of symptoms (day vs. night)?
• Is there a seasonal component to the worsening of symptoms?
• Possible triggers (viral infections, animal exposures, pollens, tobacco smoke, emotion)?
• Severity of symptoms (often reflected by unscheduled physician appointments at a walk-in clinic or emergency room, hospital admissions, and need for rescue oral corticosteroids)?
• Past investigations including chest X-rays, spirometry, allergy testing, sweat chloride testing?
• Other co-morbidities (e.g., food allergy, venom allergy)?
• Current and past treatments? Duration of use? Reasons for discontinuation?
• Barriers to treatment (cost of medication, proximity to health care providers)?
• Exposure to second- and third-hand (i.e., the lingering smell of tobacco smoke on clothing or in vehicles) tobacco smoke?
• Presence of household pets?
• Impact of the symptoms on the patient/family quality of life (missed time from activities, school or work due to asthma symptoms)?

A positive family history of asthma or other atopic diseases and/or a personal history of atopic disorders, particularly allergic rhinitis, can also be helpful in identifying patients with asthma. During the history, it is also important to enquire for possible triggers of asthma symptoms, such as cockroaches, animal dander, moulds, pollens, exercise, and exposure to tobacco smoke or cold air. When possible, objective testing for these triggers should be performed. Exposure to agents encountered in the work environment can also cause asthma. If work-related asthma is suspected, details of work exposures and improvements in asthma symptoms during holidays should be explored. It is also important to assess for comorbidities that can aggravate asthma symptoms, such as allergic rhinitis, sinusitis, obstructive sleep apnea and gastroesophageal reflux disease [16].

The diagnosis of asthma in children is often more difficult since episodic wheezing and cough are commonly associated with viral infections, and children can be asymptomatic with normal physical examinations between exacerbations. In addition, spirometry is often unreliable in patients under 6 years of age, although it can be performed in some children as young as 5 years. A useful method of confirming the diagnosis in young children is a trial of treatment (8–12 weeks of a daily ICS and a short-acting bronchodilator as needed for rescue medication). Marked clinical improvement during the treatment period, as reflected by a reduction in daytime or nocturnal symptoms of asthma, a reduction in the use of rescue bronchodilator medication, absence of acute care visits (e.g., same-day physician appointments or emergency room visits) and hospitalizations for asthma exacerbations, and the absence of rescue oral corticosteroids are all indicators that the daily ICS therapy is working and that a diagnosis of asthma is likely [5, 18, 19]. In a young child who is symptomatic with cough, wheeze, or increased difficulty breathing, a physical examination both before and after administration of a bronchodilator is of extreme value and can be used as a diagnostic tool. If the respiratory symptoms resolve within 10–15 min of bronchodilator administration, a diagnosis of asthma may be established by a physician or other healthcare provider.

The modified Asthma Predictive Index (mAPI) is a useful tool for identifying young children with recurrent wheeze who may be at high risk of developing asthma (see Table 3; also available online at: https://www.mdcalc.com/modified-asthma-predictive-index-mapi). A positive mAPI in the preschool years has been found to be highly predictive of future school-age asthma [20].

**Table 3:** Modified Asthma Predictive Index [20]

≥4 wheezing episodes in a year	
*AND*	
**At least 1 major criteria:**	*OR* **at least 2 minor criteria:**
• Parental physician-diagnosed asthma	• Wheezing unrelated to colds
• Physician-diagnosed atopic dermatitis	• Eosinophils ≥ 4% in circulation
• Allergic sensitization to at least 1 aeroallergen	• Allergic sensitization to milk, egg, or peanuts

### Physical examination

Given the variability of asthma symptoms, the physical examination of patients with suspected asthma can often be unremarkable. Physical findings may only be evident if the patient is symptomatic. Therefore, the absence of physical findings does not exclude a diagnosis of asthma. The most common abnormal physical findings are a prolonged expiratory phase and wheezing on auscultation, which confirm the presence of airflow limitation [5]. Auscultating the chest before and after bronchodilator treatment can be informative as well, with improved breath sounds noted once the small airways undergo bronchodilation.

Among children with asthma, persistent cough is also a positive finding on physical examination since not all children with asthma wheeze. Physicians should also examine the upper respiratory tract (nose, pharynx) and skin for signs of concurrent atopic conditions such as allergic rhinitis, dermatitis, and nasal polyps (also seen in cystic fibrosis) [16].

In pediatric patients, a scoring rubric called the Pediatric Respiratory Assessment Measure (PRAM) has been developed to assess a patient’s acute asthma severity using a combination of scalene muscle contraction, suprasternal retractions, wheezing, air entry and oxygen saturation (see Table 4) [21, 22]. This tool has been validated in children 0–17 years of age, and is most commonly used in acute care settings such as emergency departments, pediatric intensive care units and inpatient units.

**Table 4:** PRAM scoring table [21, 22]

Criterion	Description	Score	
O_2_ saturation (%)	≥ 95	0	
	92–94	1	
	< 92	2	
Suprasternal retraction	Absent	0	
	Present	2	
Scalene muscle	Absent	0	
contraction	Present	2	
Air entry^a^	Normal	0	
	↓ at the base	1	
	↓ at the apex and the base	2	
	Minimal or absent	3	
Wheezing^b^	Absent	0	
	Expiratory only	1	
	Inspiratory (± expiratory)	2	
	Audible without stethoscope or silent chest (minimal or no air entry)	3	
**PRAM score: (max. 12)**			

**Score**	0–3	4–7	8–12
**Severity**	Mild	Moderate	Severe

On-line tool is available at https://www.mdcalc.com/pediatric-respiratory-assessment-measure-pram-asthma-exacerbation-severity

*PRAM* Pediatric Respiratory Assessment Measure, *RUL* right upper lobe, *RML* right middle lobe, *RLL* right lower lobe, *LUL* left upper lobe, *LLL* left lower lobe, *O*_*2*_ oxygen

^a^In case of asymmetry, the most severely affected (apex-base) lung field (right or left, anterior or posterior) will determine the rating of the criterion

^b^In case of asymmetry, the two most severely affected auscultation zones, irrespectively of their location (RUL, RML, RLL, LUL, LLL), will determine the rating of the criterion

### Objective measurements to confirm variable expiratory airflow limitation

In a patient with typical respiratory symptoms, obtaining objective evidence of excessive variability in expiratory airflow limitation is essential to confirming the diagnosis of asthma (see Table 1) [5]. The greater the variations in lung function, or the more times excess variation is seen, the more likely the diagnosis is to be asthma. Spirometry is the preferred objective measure to assess for airflow limitation and excessive variability in lung function. It is recommended for all patients over 6 years of age who are able to undergo lung function testing [5, 15].

Spirometry measures airflow parameters such as the forced vital capacity (FVC, the maximum volume of air that can be exhaled) and the forced expiratory volume in 1 s (FEV_1_). Lung volumes are not measured with spirometry, and instead require full pulmonary function testing. The ratio of FEV_1_ to FVC provides a measure of airflow obstruction. In the general population, the FEV_1_/FVC ratio is usually greater than 0.75–0.80 in adults, and 0.90 in children. Any values less than these suggest airflow limitation and support a diagnosis of asthma [5, 23]. Because of the variability of asthma symptoms, patients will not exhibit reversible airway obstruction at every visit and a negative spirometry result does not rule out a diagnosis of asthma. This is particularly true for children who experience symptoms predominantly with viral infections, or who are well controlled on asthma medications. Therefore, to increase sensitivity, spirometry should be repeated, particularly when patients are symptomatic [15, 16].

Once airflow obstruction has been confirmed, obtaining evidence of excessive variability in expiratory lung function is an essential component of the diagnosis of asthma. In general, an increase in FEV_1_ of > 12% and, in adults, a change of > 200 mL from baseline after administration of a rapid-acting bronchodilator is accepted as being consistent with asthma [5, 23]. Other criteria for demonstrating excessive variability in expiratory lung function are listed in Table 1.

Spirometry must be performed according to standardized protocols (such as those proposed by the American Thoracic Society) by trained personnel. It is commonly performed in pulmonary function laboratories, but can also be performed in the outpatient clinical setting. During spirometry, the patient is instructed to take the deepest breath possible and then to exhale hard and fast and as fully as possible into the mouthpiece of the spirometer for a total of 6 s. Calibration of the spirometer should be performed daily.

Peak expiratory flow (PEF) monitoring is an acceptable alternative *when spirometry is not available*, and can also be useful for diagnosing occupational asthma and/or monitoring response to asthma treatments. However, PEF is not recommended for diagnosing asthma in children. PEF is usually measured in the morning and in the evening. A diurnal variation in PEF of more than 20% or an improvement of at least 60 L/min or at least 20% after inhalation of a rapid-acting bronchodilator suggests asthma [15]. Although simpler to perform than spirometry, PEF is more effort-dependent and much less reliable. Therefore, as mentioned earlier, spirometry is the preferred method of documenting variable expiratory airflow limitation and confirming the diagnosis of asthma.

The importance of objective measures for confirming the diagnosis of asthma cannot be overemphasized. The results of a recent multicentre study that included 613 adults with physician-diagnosed asthma from across Canada found that the diagnosis of current asthma was ruled out in 33% of patients; these subjects were not using daily asthma medications or had been weaned off medication [24]. Compared to subjects whose current asthma diagnosis was confirmed, those in whom the diagnosis was ruled out were less likely to have undergone testing for airflow limitation in the community at the time of the initial diagnosis. These findings suggest that re-evaluation of an asthma diagnosis may be warranted.

### Tests of bronchial hyperreactivity

When spirometry is normal, but symptoms and the clinical history are suggestive of asthma, measurement of airway responsiveness using direct airway challenges to inhaled bronchoconstrictor stimuli (e.g., methacholine or histamine) or indirect challenges (e.g., with mannitol or exercise) may help confirm a diagnosis of asthma.

Tests of bronchial hyperreactivity should be conducted in accordance with standardized protocols in a pulmonary function laboratory or other facility equipped to manage acute bronchospasm. Bronchopovocation testing involves the patient inhaling increasing doses or concentrations of an inert stimulus until a given level of bronchoconstriction is achieved, typically a 20% fall in FEV_1_. An inhaled rapid-acting bronchodilator is then provided to reverse the obstruction. Test results are usually expressed as the provocative dose (PD) or provocative concentration (PC) of the provoking agent that causes the FEV_1_ to drop by 20% (the PD_20_ or PC_20_, respectively). For methacholine, most pulmonary function laboratories use a PC_20_ value less than 4-8 mg/mL as the threshold for a positive result indicative of airway hyperreactivity, supporting a diagnosis of asthma. However, positive challenge tests are not specific to asthma and may occur with other conditions such as allergic rhinitis and chronic obstructive pulmonary disease (COPD). Therefore, tests of bronchial hyperreactivity may be most useful for ruling out asthma among individuals who are symptomatic. A negative test result in a symptomatic patient not receiving anti-inflammatory therapy is highly sensitive [16].

In order to properly assess lung function, patients who have been prescribed a combination of an ICS and a LABA must discontinue these long-acting medications 24 h prior to tests of airway hyperreactivity or testing with spirometry. Tests of bronchial hyperreactivity are contraindicated in patients with FEV_1_ values less than 60–70% of the normal predicted value (since bronchoprovocation could cause significant bronchospasm), in patients with uncontrolled hypertension or in those who recently experienced a stroke or myocardial infarction [25].

### Non-invasive markers of airway inflammation

The measurement of inflammatory markers such as sputum eosinophilia (proportion of eosinophils in the cell analysis of sputum) or levels of exhaled nitric oxide (a gaseous molecule produced by some cells during an inflammatory response) can also be useful for diagnosing asthma. Evidence suggests that exhaled nitric oxide levels can be supportive of the diagnosis of asthma, and may also be useful for monitoring patient response to asthma therapy [16]. It is still not accepted as a standard test for the diagnosis of asthma. Although these tests have been studied in the diagnosis and monitoring of asthma, they are not yet widely available in Canada.

### Allergy skin testing

Allergy skin prick (epicutaneous) testing is recommended to identify possible environmental allergic triggers of asthma, and is helpful in identifying the asthma phenotype of the patient. Testing is typically performed using the allergens relevant to the patient’s geographic region. Although allergen-specific IgE tests that provide an in vitro measure of a patient’s specific IgE levels for specific allergens have been suggested as an alternative to skin tests, these tests are less sensitive, more invasive (requires venipuncture), and more expensive than skin prick tests [5, 15]. There is no minimum age at which skin prick testing can be performed.

### Differential diagnosis

Conditions that should be considered in the differential diagnosis of adults with suspected asthma may include: COPD, bronchitis, gastrointestinal reflux disease, recurrent respiratory infections, heart disease, and vocal cord dysfunction. Distinguishing asthma from COPD can be particularly difficult as some patients have features of both disorders. The term asthma-COPD overlap syndrome (ACOS), though not a single disease entity, has been adopted to describe these patients. A recent population-based cohort study conducted in Ontario suggests that the prevalence of concurrent asthma and COPD is increasing, particularly in women and young adults [26].

The differential diagnosis of asthma is unique for infants and young children and includes anatomic defects (laryngo- or tracheomalacia, congenital heart defects), physiological defects (primary ciliary dyskinesia) and genetic conditions such cystic fibrosis and primary immunodeficiency, to name just a few conditions. A chest X-ray may be considered in the work-up of a child with suspected asthma, particularly if the diagnosis is unclear or if the child is not responding as expected to treatment. Table 5 lists conditions to consider in the differential diagnosis of recurrent respiratory symptoms in children.

**Table 5:** Differential diagnosis of recurrent respiratory symptoms in children [31, 36]

**Infections**	**Congenital problems**
• Recurrent respiratory tract infections • Chronic rhino-sinusitis • Tuberculosis	• Tracheomalacia • Tracheo-esophageal fistula • Cystic fibrosis • Bronchopulmonary dysplasia
**Mechanical problems** • Foreign body aspiration • Gastroesophageal reflux • Vocal cord dysfunction	• Congenital malformation causing narrowing of the intrathoracic airways • Primary ciliary dyskinesia syndrome • Immune deficiency • Congenital heart disease

## Management

**Fig. 1:**
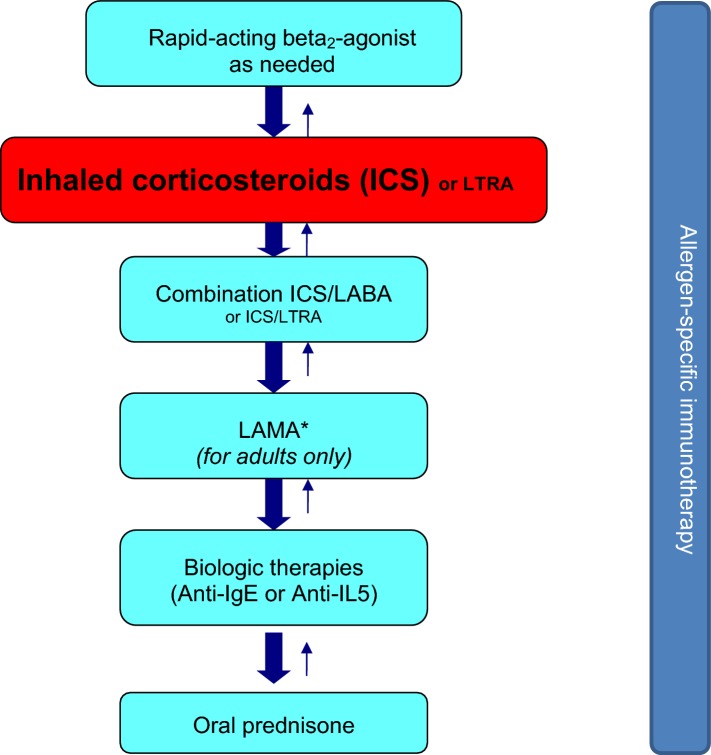
**A simplified, stepwise algorithm for the treatment of asthma.** *LAMAs are not indicated in persons < 18 years of age. *ICS* inhaled corticosteroid, *LTRA* leukotriene receptor antagonist, *LABA* long-acting beta_2_-agonist, *IgE* immunoglobulin E, *IL-5* interleukin 5; *LAMA* long-acting muscarinic receptor antagonist. **Note: Treatments can be used individually or in any combination**

The primary goal of asthma management is to achieve and maintain control of the disease in order to prevent exacerbations (abrupt and/or progressive worsening of asthma symptoms that often require immediate medical attention and/or the use of oral steroid therapy) and reduce the risk of morbidity and mortality. Other goals of therapy are to minimize the frequency and severity of asthma symptoms, decrease the need for reliever medications, normalize physical activity, and improve lung function as well as overall quality of life. The level of asthma control should be assessed at each visit using the criteria in Table 6, and treatment should be tailored to achieve control. In most asthma patients, control can be achieved using both trigger avoidance measures and pharmacological interventions. The pharmacologic agents commonly used for the treatment of asthma can be classified as controllers (medications taken daily on a long-term basis that achieve control primarily through anti-inflammatory effects) and relievers (medications used on an as-needed basis for quick relief of bronchoconstriction and symptoms). Controller medications include ICSs, leukotriene receptor antagonists (LTRAs), LABAs in combination with an ICS, long-acting muscarinic receptor antagonists (LAMAs), and biologic agents including anti-IgE therapy and anti-IL-5 therapy. Reliever medications include rapid-acting inhaled beta_2_-agonists and inhaled anticholinergics [5, 15, 16]. Allergen-specific immunotherapy may also be considered in most patients with allergic asthma, but must be prescribed by physicians who are adequately trained in the treatment of allergies (see* Allergen-specific immunotherapy* article in this supplement) [27–30]. Systemic corticosteroid therapy may also be required for the management of acute asthma exacerbations. A simplified, stepwise algorithm for the treatment of asthma is provided in Fig. 1.

**Table 6:** Criteria for assessing asthma control [5, 15]

No exacerbations
Fewer than 3 doses/week of a rapid-acting beta_2_-agonist bronchodilator
Daytime symptoms < 3 days/week
No nighttime symptoms
Normal physical activity
No absenteeism from work or school
FEV_1_ or PEF at least 90% of personal best

*FEV*_*1*_ forced expiratory volume in 1 s, *PEF* peak expiratory flow

The goal of asthma therapy is to treat individuals using the least amount of medications required to control asthma symptoms and maintain normal daily activities. When asthma control has been achieved, ongoing monitoring and follow-up are essential to monitor for side effects, preserve lung function over time, observe for new triggers, and establish the minimum maintenance doses required to maintain control. However, because asthma is a variable disease, treatment may need to be adjusted periodically in response to loss of control (as indicated by failure to meet the control criteria in Table 6) [5]. It is also imperative that all asthma patients be empowered to take an active role in the management of their disease. This can be accomplished by providing patients with a personalized written action plan for disease management and by educating the patient about the nature of the disease, the role of medications, the importance of adhering to controller therapy, and the appropriate use of inhaler devices [16]. Once a written action plan for management is provided, ongoing follow up should include:

- Reviewing the asthma action plan at each visit to determine if modifications are required based on level of asthma control;
- Observation of inhaler device technique at each visit;
- Counselling patients or caregivers who smoke on smoking cessation;
- Measuring height and weight of children and adolescents to monitor growth velocity and potential corticosteroid side effects;
- Screening for signs and symptoms of adrenal suppression for individuals requiring moderate- to high-dose ICS;
- Asking about food or venom allergies and ensuring that patients with these allergies are prescribed an epinephrine autoinjector and provided with a written anaphylaxis plan. Patients with poorly controlled asthma and food/venom allergy are at greater risk for anaphylaxis upon accidental exposure to their known allergen (see* Anaphylaxis* article in this supplement).
- Referring individuals who have difficulty achieving asthma control to an asthma specialist (respirologist, allergist or certified asthma educator) for further assessment (see “Indications for referral” section in this article).

### Avoidance measures

Avoidance of exposure to tobacco smoke is important for all patients with asthma. Avoidance of other relevant allergens/irritants is also an important component of asthma management. Patients allergic to house dust mites should be instructed to use allergen-impermeable covers for bedding and to keep the relative humidity in the home below 50% (to inhibit mite growth). Pollen exposure can be reduced by keeping windows closed, using an air conditioner, and limiting the amount of time spent outdoors during peak pollen seasons. For patients allergic to animal dander, removal of the animal from the home is recommended and usually results in a significant reduction in symptoms within 4–6 months. However, compliance with this recommendation is poor and, therefore, the use of high-efficiency particulate air (HEPA) filters and restricting the animal from the bedroom or to the outdoors may be needed to help decrease allergen levels. Measures for reducing exposure to mould allergens include cleaning with fungicides, de-humidification to less than 50%, and HEPA filtration [16].

Since these avoidance strategies can be labour-intensive, patient adherence is usually suboptimal. Frequent reassessments, encouragement and empowerment by the treating physician are often required to help promote adherence to these strategies. Furthermore, patients should be advised to use a combination of avoidance measures for optimal results, since single-strategy interventions have demonstrated no measurable benefits in asthma control [16].

### Inhaled medication delivery devices

Inhaled asthma medications come in a variety forms including pressurized metered-dose inhalers (pMDIs) and dry powder inhalers (DPIs) (Turbuhaler, Diskus, Twisthaler, Ellipta). Not all medications are available in the same delivery devices. Also, some devices have dose counters included and others, such as pMDIs, do not. The most important factor in selecting a medication delivery device is to ensure that the patient uses it properly.

In children, it is recommended that pMDIs always be used with a spacer device since they are as effective as nebulizers; a pMDI with spacer is also preferred over nebulizers [31]. A spacer with face mask is recommended for children 2–4 years of age, while a spacer with mouthpiece is recommended for children 4–6 years of age. To transition to a spacer with mouthpiece, children must be able to form a seal around the mouthpiece and breathe through their mouths. For children 6 years of age or over, a pMDI plus spacer with mouthpiece or DPI is recommended. Since children must have sufficient inspiratory force to use a DPI, these devices are generally not recommended for children under 6 years of age.

### Reliever medications

Inhaled rapid-acting beta_2_-agonists are the preferred reliever medications for the treatment of acute symptoms, and should be prescribed to all patients with asthma. In Canada, several short-acting beta_2_-agonists (SABAs; e.g., salbutamol, terbutaline) and one LABA (formoterol) are approved for this indication. SABAs should only be taken on an as needed basis for symptom relief. Use of an as-needed SABA in the absence of a controller therapy should be reserved for patients with symptoms less than twice per month, without nocturnal wakening in the past month, or an exacerbation within the past year. In children with well controlled asthma, a SABA should be used less than three times per week.

Unlike other LABAs, formoterol has a rapid onset of action and, therefore, can be used for acute symptom relief. Given that LABA monotherapy has been associated with an increased risk of asthma-related morbidity and mortality, formoterol should only be used as a reliever in patients 12 years of age or older who are on regular controller therapy with an ICS [5, 15, 16, 23].

Short-acting anticholinergic bronchodilators, such as ipratropium bromide, may also be used as reliever therapy. These agents appear to be less effective than inhaled rapid-acting beta_2_-agonists and, therefore, should be reserved as second-line therapy for patients who are unable to use SABAs. They may also be used in addition to SABAs in patients experiencing moderate to severe asthma exacerbations. Short-acting anticholinergic bronchodilator therapy is not recommended for use in children [15].

### Controller medications

#### Inhaled corticosteroids (ICSs)

ICSs are the most effective anti-inflammatory medications available for the treatment of asthma and represent the mainstay of therapy for most patients with the disease. Low-dose ICS monotherapy is recommended as first-line maintenance therapy for most children and adults with asthma. Regular ICS use has been shown to reduce symptoms and exacerbations, and improve lung function and quality of life. ICSs do not, however, “cure” asthma, and symptoms tend to recur within weeks to months of ICS discontinuation. Most patients will require long-term, if not life-long, ICS treatment [5, 15, 16].

Since ICSs are highly effective when used optimally, factors other than treatment efficacy need to be considered if ICS therapy is unsuccessful in achieving asthma control. These factors include: misdiagnosis of the disease, poor adherence to ICS therapy, improper inhaler technique, continued trigger exposure or the presence of other comorbidities. If, after addressing such factors, patients fail to achieve control with low-to-moderate ICS doses, then treatment should be modified. For most children, ICS dose escalation (to a moderate dose) is the preferred approach to achieve control, while the addition of another class of medications (usually a LABA) is recommended for patients over 12 years of age [15, 16, 23]. Low, medium and high doses of ICS therapy varies by age and are summarized in Table 7. Children who fail to achieve control on a moderate ICS dose should be referred to an asthma specialist, such a respirologist, an allergist, an immunologist or a pediatrician. It is also recommended that children receiving daily ICS therapy do not increase their daily ICS dose with the onset of a viral illness [23].

**Table 7:** Overview of the main controller therapies used for the treatment of asthma [23, 31]

	Usual adult dose	Pediatric dose information	
		< 6 years of age	6–18 years of age
**ICSs**			
Beclomethasone (Qvar, generics)	pMDI: 100–800 µg/day, divided bid	pMDI • *Low* 50 µg bid • *Med* 100 µg bid • *High* refer to specialist *Approved age by Health Canada *≥* 5* *years*	pMDI • *Low* 50–100 µg bid • *Med* > 100 µg bid • *High* > 200 µg bid
Budesonide (Pulmicort)	DPI: 400–2400 µg/day, divided bid Nebules: 1–2 mg bid	DPI not recommended for children < 6 years	DPI • *Low* 100 µg bid • *Med* 200–400 µg bid • *High* > 400 µg bid *Approved age by Health Canada* ≥* 6* *years*
		Nebules: 0.25–0.5 mg bid (for children 3 months to 12 years)	
Ciclesonide (Alvesco)	pMDI: 100–800 µg/day	pMDI: • *Low* 100 µg once daily • *Med* 200 µg daily • *High* refer to specialist	pMDI: • *Low* 100 µg once daily • *Med* 200–400 µg daily • *High* > 400 µg daily *Approved age by Health Canada* ≥* 6* *years*
Fluticasone propionate (Flovent HFA, Flovent Diskus)	pMDI/DPI: 100–500 µg bid	pMDI/DPI • *Low* 50 µg bid • *Med* 100–125 µg bid • *High* refer to specialist *Approved age by Health Canada *≥* 1* *year for pMDI, *≥* 4* *years for Diskus (DPI)*	pMDI/DPI • *Low* ≤ 100 µg bid • *Med* > 100–200 µg bid • *High* ≥ 200 µg bid
Mometasone (Asmanex)	DPI: 200–400 µg/day	DPI not recommended for children <6 years	DPI • *Low* ≤ 200 µg daily • *Med* > 100–200 µg bid • *High* > 200 µg bid *Approved age by Health Canada* ≥ *12* *years*
Fluticasone furoate (Arnuity Ellipta)	DPI: 100–200 µg/day	Not indicated for children < 12 years	
**Combination ICS/LABA inhalers**			
Budesonide/formoterol (Symbicort)	DPI (maintenance): 100/6 µg or 200/6 µg, 1–2 puffs od or bid; max 4 puffs/day DPI (maintenance and reliever): 100/6 µg or 200/6 µg, 1–2 puffs bid or 2 puffs od; plus 1 puff prn for relief of symptoms (no more than 6 puffs on any single occasion); max 8 puffs/day	Refer to specialist	DPI • *Low* 100/6 µg 1 dose bid • *Med* 100/6 µg 2 doses bid, 200/6 µg 1–2 doses bid • *High* > 200/6 µg 2 doses bid *Approved age by Health Canada* ≥ *12* *years*
Fluticasone furoate/salmeterol (Advair pMDI, Advair Diskus)	pMDI: 125/25 µg or 250/25 µg, 2 puffs bid Diskus: 100/50 µg, 250/50 µg or 500/50 µg: 1 puff bid	Refer to specialist *Approved age by Health Canada *≥* 4* *years for Diskus (DPI)*	DPI/pMDI • *Low* 100/50 µg bid • *Med* > 100–200 µg bid • *High* ≥ 250/50 µg bid *Approved age by Health Canada *≥* 12* *years for pMDI*
Mometasone/formoterol (Zenhale)	For patients previously treated with Low-dose ICS: 50/5 µg, 2 puffs bid Medium-dose ICS: 100/5 µg, 2 puffs bid High-dose ICS: 200/5 µg, 2 puffs bid	Refer to specialist	pMDI • *Low* 50/5–100/5 μg 1 dose bid • *Med* 100/5 μg 2 doses bid, 200/5 μg 1–2 doses bid • *High* > 200/5 μg *Approved age by Health Canada* ≥* 12* *years*
Fluticasone furoate/vilanterol (Breo Ellipta)	DPI: 100/25 µg/day or 200/25 µg/day	Not indicated for children < 18 years of age	
**LTRAs**			
Montelukast (Singulair)	10 mg tablet od (taken in the evenings)	4 mg po daily *Approved age by Health Canada* ≥*2* *years*	5 mg po daily (6–14 years) 10 mg po daily (≥ 15 years)
Zafirlukast (Accolate)	20 mg tablet bid, at least 1 h before or 2 h after meals	Refer to specialist	20 mg tablet bid, at least 1 h before or 2 h after meals *Approved age by Health Canada *≥* 12* *years*
**LAMAs**			
Tiotropium (Spiriva Respimat)	1.25 µg, 2 puffs od	Not indicated for children < 18 years	
**Anti-IgE therapy**			
Omalizumab (Xolair)	150–375 mg sc every 2–4 weeks (based on patient’s weight and pre-treatment serum IgE level)	Not indicated for children < 6 years	75–375 mg sc every 2–4 weeks (based on patient’s weight and pre-treatment serum IgE level)
**Anti-IL5 therapy**			
Mepolizumab (Nucala)	100 mg sc every 4 weeks	Not indicated for children < 18 years	
Reslizumab (Cinqair)	3 mg/kg IV every 4 weeks	Not indicated for children < 18 years	
Benralizumab (Fasenra)	30 mg sc every 4 weeks for the first 3 doses, then every 8 weeks thereafter	Not indicated for children < 18 years	

Pediatric dose information adapted from BCGuidelines.ca Guidelines & Protocols Advisory Committee, 2015 [31]

*ICS* inhaled corticosteroid, pMDI pressurized metered-dose inhaler, *DPI* dry powder inhaler, *LTRA* leukotriene receptor antagonists, *IgE* immunoglobulin E, *IL-5* interleukin 5, *bid* twice daily, *sc* subcutaneously, *IV* intravenously, *LABA* long acting beta agonist, *LAMA* long-acting muscarinic receptor antagonist, *po* oral, *prn* as needed

*Side effects* The most common local adverse events associated with ICS therapy are oropharyngeal candidiasis (also known as oral thrush) and dysphonia (hoarseness, difficulty speaking). Rinsing and expectorating (spitting) after each treatment and the use of a spacer with pMDI devices can help reduce the risk of these side effects. Systemic adverse effects with ICS therapy are rare, but may occur at high doses, such as > 500 μg of fluticasone propionate equivalent, and include changes in bone density, cataracts, glaucoma and growth retardation [5]. Patients using high ICS doses should also be monitored for adrenal suppression [32]. It is important to note that the potential for side effects with ICS therapy needs to be considered in the context of other steroids (i.e., systemic, intranasal and topical) that may be prescribed for other atopic conditions such as allergic rhinitis or atopic dermatitis.

#### Combination ICS/LABA inhalers

LABA monotherapy is not recommended in patients with asthma as it does not impact airway inflammation and is associated with an increased risk of morbidity and mortality. LABAs are only recommended when used in combination with ICS therapy. The combination of a LABA and ICS has been shown to be highly effective in reducing asthma symptoms and exacerbations, and is the preferred treatment option in adolescents or adults whose asthma is inadequately controlled on low-dose ICS therapy, or in children over 6 years of age who are uncontrolled on moderate ICS doses [15, 23]. Although there is no apparent difference in efficacy between ICSs and LABAs given in the same or in separate inhalers, combination ICS/LABA inhalers are preferred because they preclude use of the LABA without an ICS, are more convenient and may enhance patient adherence. Four combination ICS/LABA inhalers are available in Canada: fluticasone propionate/salmeterol, budesonide/formoterol, mometasone/formoterol and fluticasone furoate/vilanterol (see Table 7). Combination budesonide/formoterol has been approved for use as a single inhaler for both daily maintenance (controller) and reliever therapy in individuals 12 years of age and older. It should only be used in patients whose asthma is not adequately controlled with low-dose ICS who warrant treatment with combination therapy [5, 15, 23].

#### Leukotriene receptor antagonists

The LTRAs, montelukast and zafirlukast, are also effective for the treatment of asthma and are generally considered to be safe and well tolerated. Because these agents are less effective than ICS treatment when used as monotherapy, they are usually reserved for patients who are unwilling or unable to use ICSs. LTRAs can also be used as add-on therapy if asthma is uncontrolled despite the use of low-to-moderate dose ICS therapy or combination ICS/LABA therapy. It is important to note, however, that LTRAs are considered to be less effective than LABAs as add-on therapy in adults [5, 15, 23]. In children, if medium-dose ICS therapy is ineffective, LTRAs are considered the next-line treatment option [23]. If, however, the child has persistent airway obstruction, the addition of a LABA may be preferred.

#### Long-acting muscarinic receptor antagonists

The LAMA, tiotropium, administered by mist inhaler can be used as add-on therapy for patients with a history of exacerbations despite treatment with ICS/LABA combination therapy. It is only indicated for patients 12 years of age and older.

#### Theophylline

Theophylline is an oral bronchodilator with modest anti-inflammatory effects. Given its narrow therapeutic window and frequent adverse events (e.g., gastrointestinal symptoms, loose stools, seizures, cardiac arrhythmias, nausea and vomiting), its use is generally reserved for patients over 12 years of age who are intolerant to or continue to be symptomatic despite other add-on therapies [5, 15].

#### Biologic therapies

The anti-IgE monoclonal antibody, omalizumab, has been shown to reduce the frequency of asthma exacerbations by approximately 50%. The drug is administered subcutaneously once every 2–4 weeks and is approved in Canada for the treatment of moderate to severe, persistent allergic asthma in patients 6 years of age or older. At present, omalizumab is reserved for patients with difficult to control asthma who have documented allergies, an elevated serum IgE level, and whose asthma symptoms remain uncontrolled despite ICS therapy in combination with a second controller medication [15].

Two monoclonal antibodies to IL-5 have been approved in Canada for patients aged 18 years or older with severe eosinophilia: mepolizumab and reslizumab. These are given every 4 weeks by subcutaneous injection and intravenous infusion, respectively, and are indicated in patients who are uncontrolled despite treatment with high-dose ICS therapy and an additional controller therapy, such as a LABA, and who have elevated blood eosinophils [5]. Recently, benralizumab, a monoclonal antibody against the IL-5 receptor has also been approved in Canada for the treatment of adult patients with severe eosinophilic asthma.

Table 7 provides a list of the commonly used controller therapies and their recommended dosing regimens. It is important to note that long-term compliance with controller therapy is poor because patients tend to stop therapy when their symptoms subside. Therefore, regular follow-up visits are important to help promote treatment adherence.

### Systemic corticosteroids

Systemic corticosteroids, such as oral prednisone, are generally used for the acute treatment of moderate to severe asthma exacerbations. While chronic systemic corticosteroid therapy may also be effective for the management of difficult to control asthma, prolonged use of oral steroids are associated with well-known and potentially serious adverse effects and, therefore, their routine or long-term use should be avoided if at all possible, particularly in children [23]. Adverse events with short-term, high-dose oral prednisone are uncommon, but may include: reversible abnormalities in glucose metabolism, increased appetite, edema, weight gain, rounding of the face, mood alterations, hypertension, peptic ulcers and avascular necrosis of the hip [5].

### Bronchial thermoplasty

Bronchial thermoplasty involves the treatment of airways with a series of radiofrequency pulses. This treatment may be considered for adult patients with severe asthma despite pharmacotherapy [5].

### Allergen-specific immunotherapy

Allergen-specific immunotherapy involves the subcutaneous or sublingual administration of gradually increasing quantities of the patient’s relevant allergens until a dose is reached that is effective in inducing immunologic tolerance to the allergen. Although it has been widely used to treat allergic asthma, it is not universally accepted by all clinical practice guideline committees due to the potential for serious anaphylactic reactions with this form of therapy [28].

A Cochrane review of 88 randomized controlled trials examining the use of allergen-specific immunotherapy in asthma management confirmed its efficacy in reducing asthma symptoms and the use of asthma medications, and improving airway hyperresponsiveness [27]. Similar benefits have been noted with sublingual immunotherapy [33], which is now available for use in Canada for grass and ragweed allergies, as well as house dust mite-induced allergic rhinitis (see* Allergen-specific immunotherapy* article in this supplement). Evidence also suggests that allergen-specific immunotherapy may prevent the onset of asthma in atopic individuals [34, 35].

At present, allergen-specific immunotherapy should be considered on a case-by-case basis. Allergen-specific subcutaneous immunotherapy may be considered as add-on therapy in patients using ICS monotherapy, combination ICS/LABA inhalers, ICS/LTRAs and/or omalizumab if asthma symptoms are controlled. It should not be initiated in patients with uncontrolled asthma or an FEV_1_ < 70% of predicted. For subcutaneous immunotherapy, asthma must be controlled at the time of each injection, and it must be administered in clinics that are equipped to manage possible life-threatening anaphylaxis where a physician is present. Since allergen-specific immunotherapy carries the risk of anaphylactic reactions, it should only be prescribed by physicians who are specialists in allergy [5].

### Indications for referral

In older children, adolescents and adults, referral to a specialist in asthma care (e.g., respirologist, allergist) is recommended when:

- Atypical asthma symptoms are present or the diagnosis of asthma is in question;
- The patient has poor asthma control (poor lung function, persistent asthma symptoms) or severe asthma exacerbations (≥ 1 course of systemic steroids per year or hospitalization) despite moderate doses of ICS (with proper technique and good compliance);
- The patient requires a detailed assessment for and management of potential environmental triggers;
- The patient has been admitted to the intensive care unit (ICU) for asthma.

In young children 1–5 years of age, referral to an asthma specialist is recommended when there is diagnostic uncertainty or suspicion of comorbidity; poor symptom and exacerbation control despite ICS at daily doses of 200–250 µg; a life-threatening event (requiring ICU admission and/or intubation); and/or for allergy testing to assess the possible role of environmental allergens [17].

## Conclusion

Asthma is the most common respiratory disorder in Canada, and contributes to significant morbidity and mortality. A diagnosis of asthma should be suspected in patients with recurrent cough, wheeze, chest tightness and dyspnea, and should be confirmed using objective measures of lung function (spirometry preferred). Allergy testing is also recommended to identify possible triggers of asthma symptoms.

In most patients, asthma control can be achieved using avoidance measures and appropriate pharmacological interventions. ICSs represent the standard of care for the majority of asthma patients. For those who fail to achieve control with low-to-moderate ICS doses, combination therapy with a LABA and ICS is the preferred treatment choice in most adults. LTRAs can also be used as add-on therapy if asthma is uncontrolled despite the use of low-to-moderate dose ICS therapy, particularly in patients with concurrent allergic rhinitis. LAMAs or biologic therapies targeting IgE or IL-5 may be useful in select cases of difficult to control asthma. Allergen-specific immunotherapy is a potentially disease-modifying therapy, but should only be prescribed by physicians with appropriate training in allergy. All patients with asthma should have regular follow-up visits during which criteria for asthma control, adherence to therapy and proper inhaler technique should be reviewed.

## Key take-home messages

- A clinical diagnosis of asthma should be suspected in patients with intermittent symptoms of wheezing, coughing, chest tightness and breathlessness.
- Objective measurements of lung function, preferably using spirometry, are needed to confirm the diagnosis. The best time to perform this testing is when the patient is symptomatic. Spirometry can generally be performed in children 6 years of age and older.
- In children < 6 years of age who are unable to perform spirometry, a trial of therapy (8–12 weeks in duration) and monitoring of symptoms can act as a surrogate method to diagnose asthma.
- All asthma patients should be prescribed a rapid-acting bronchodilator to be used as needed for relief of acute symptoms.
- ICS therapy is the standard of care for most patients with asthma.
- Combination ICS/LABA inhalers are recommended for most adult patients who fail to achieve control with low-to-moderate ICS doses.
- LTRAs can also be used as add-on therapy if asthma is uncontrolled despite the use of low-to-moderate ICS doses.
- Tiotropium by mist inhaler can be added in patients 12 years of age or older with an exacerbation history despite ICS/LABA treatment.
- Biologic therapy targeting IgE or IL-5 may be useful in select cases of difficult to control asthma.
- Allergen-specific immunotherapy is a potentially disease-modifying therapy that can be considered in most cases of allergic asthma.
- Regular monitoring of asthma control every 3–4 months, adherence to therapy and inhaler technique are important components of asthma management.

## Abbreviations

ICS: inhaled corticosteroid
LABA: long-acting beta_2_-agonist
IgE: immunoglobulin E
IL: interleukin
GINA: Global Initiative for Asthma
Th2: T helper cell type-2
BMI: body mass index
FEV_1_: forced expiratory volume in one second
FVC: forced vital capacity
LTRA: leukotriene receptor antagonist
LAMA: long-acting muscarinic receptor antagonist
pMDI: pressurized metered dose inhalers
DPI: dry powder inhaler
PD: provocative dose
PC: provocative concentration
PEF: peak expiratory flow
COPD: chronic obstructive pulmonary disease
ACOS: asthma-COPD overlap syndrome
CHILD: Canadian Healthy Infant Longitudinal Development
SARP: Severe Asthma Research Program
PRAM: Pediatric Respiratory Assessment Measure
HEPA: high-efficiency particulate air
SABA: short-acting beta_2_-agonist
ICU: intensive care unit
RSV: respiratory syncytial virus
mAPI: modified Asthma Predictive Index
